# Cardiac metastases from neuroendocrine neoplasms: complementary role of SSTR PET/CT and cardiac MRI

**DOI:** 10.1007/s12350-023-03345-w

**Published:** 2023-08-16

**Authors:** Evyn G. Arnfield, Laura Tam, David A. Pattison, John Younger, Venkata Avinash Chikatamarla, David Wyld, Matthew Burge, Louise McCormack, Rahul Ladwa, Stuart Ramsay

**Affiliations:** 1 Department of Nuclear Medicine & Specialised PET Services, Royal Brisbane and Women’s Hospital, Brisbane, QLD 4006 Australia; 2 Faculty of Medicine, University of Queensland, Brisbane, Australia; 3 Department of Medical Oncology, Princess Alexandra Hospital, Woolloongabba, Australia; 4 Department of Cardiology, Royal Brisbane and Women’s Hospital, Brisbane, Australia; 5 Department of Medical Oncology, Royal Brisbane and Women’s Hospital, Brisbane, Australia

**Keywords:** PET, MRI, hybrid imaging, molecular imaging, other

## Abstract

**Background:**

Cardiac metastases from neuroendocrine neoplasms (NENs) are being detected with increasing frequency, although the optimal imaging strategy remains unclear. We performed a single-center retrospective study to explore the role of somatostatin receptor positron emission tomography/computed tomography (SSTR PET/CT) and cardiac magnetic resonance imaging (CMR) in NEN cardiac metastases, determine the degree of concordance between the findings of these imaging modalities, and examine the advantages and disadvantages of each imaging technique. A secondary aim was to determine if cardiac metastases were associated with adverse cardiac events during peptide receptor radionuclide therapy (PRRT).

**Methods and results:**

19 patients with NEN cardiac metastases were identified. A retrospective review of electronic medical records was performed, and if available SSTR PET/CT and CMR were blindly re-reviewed by imaging specialists, documenting the number and location of cardiac metastases. All 19 patients had SSTR PET/CT, and 10/19 patients had CMR. SSTR PET/CT identified more metastases than CMR. When identified on CMR, metastases were more accurately localized. 12/19 patients received PRRT, with no cardiac adverse effects.

**Conclusion:**

SSTR PET/CT and CMR are complementary investigations in the imaging of NEN cardiac metastases. SSTR PET/CT appears more sensitive for lesion detection, and CMR offers better lesion characterization. Both investigations present useful information for the planning of treatment including PRRT, which was administered safely.

**Supplementary Information:**

The online version contains supplementary material available at 10.1007/s12350-023-03345-w.

## Introduction

Neuroendocrine neoplasms (NENs) are a clinically heterogenous group of tumors arising from neuroendocrine cells, most commonly in the gastroenteropancreatic and pulmonary systems.^1^ Although most NENs have an indolent disease course, a subset are more biologically aggressive with higher proliferative rates and a propensity to metastasize.^2^ The incidence and prevalence of NENs have been rising worldwide in recent decades,^1,3–5^ with overall mortality remaining largely stable.^6^ This trend likely reflects improved diagnosis, as well as an increase in the use of therapeutic techniques such as peptide receptor radionuclide therapy (PRRT), a form of systemically administered radiation therapy which selectively targets receptors expressed on the surface of tumor cells.^5^

A key characteristic of NENs is the over-expression of somatostatin receptors, particularly somatostatin receptor 2 (SSTR2).^7^ The presence of these receptors on the surface of tumor cells facilitates detection through the use of positron emission tomography/computed tomography (PET/CT) using ^68^Ga-DOTA somatostatin analogues (DOTA-TOC/TATE/NOC) and is considered the gold-standard imaging technique for the detection and staging of NENs.^7–10^ Furthermore, demonstration of adequate density and distribution of somatostatin receptor expression on SSTR PET/CT at sites of NEN metastatic disease facilitates treatment with PRRT if clinically appropriate.^10^

The European Neuroendocrine Tumour Society (ENETS)/World Health Organisation grading system classifies NENs as grade 1, 2, or 3 based on the mitotic rate and the immunohistochemical staining of the Ki-67 protein, which is a marker of cellular proliferation. Higher grade NENs may demonstrate reduced avidity on SSTR PET/CT reflecting decreased SSTR expression and increased avidity on 2-[^18^F]fluoro-2-deoxy-d-gluocse PET/CT ([^18^F]FDG PET/CT) reflecting higher glycolytic metabolism; the so-called “flip flop” phenomenon.^10,11^ NENs can also demonstrate tumor heterogeneity within and between sites of disease throughout the body, and dual tracer molecular imaging with SSTR PET/CT and FDG PET/CT can facilitate whole body tumor characterization in a manner that targeted biopsy cannot. This has a significant management impact, with more well differentiated SSTR expressing disease usually being more suitable for PRRT, and more poorly differentiated metabolically active disease usually being more suitable for conventional treatments including chemotherapy.

Cardiac metastases from NENs have previously been considered extremely rare, however with the increased utilization of SSTR PET/CT there has been a notable rise in their detection.^7^ Current estimates of the incidence are 1%-4% of NEN patients, with a possible male predilection.^12^ They most commonly arise from well-differentiated small bowel NENs in an advanced stage of disease and are not associated with an increased incidence of carcinoid valvular disease.^12^ Cardiac metastases have also been reported from primary pulmonary^13^ and pancreatic NENs.^14^

In most instances cardiac metastases are incidental findings in clinically asymptomatic patients.^7^ Rarely, cardiac metastases may be associated with complications including life-threatening arrhythmias, cardiac failure, and ventricular outflow tract obstruction.^15^ The presence of cardiac metastases also has potential implications for the delivery of PRRT, which could induce lesion necrosis, theoretically causing derangements in cardiac structure and electrophysiology, including pericardial effusions, pericarditis, cardiac tamponade, and even cardiac rupture. Despite this, it should be noted that the only reported local complication from PRRT in patients with NEN cardiac metastases is pericarditis.^16^ Surgery is recommended for patients with symptomatic cardiac metastases,^12^ especially if the lesion causes hemodynamic compromise or valvular obstruction, and several case reports have demonstrated that surgical management for high-risk cardiac lesions may be beneficial.^17,18^

A European Neuroendocrine Tumour Society (ENETS) consensus statement briefly states that PRRT can be considered in patients with cardiac metastases,^12^ but current detailed PRRT guidelines do not make any specific mention of cardiac metastases, including the need for additional precautions or advanced cardiac imaging.^19^ A few small case reports and case series have described the use of PRRT in this patient cohort without significant cardiac complications.^16,20–24^

Several case reports have previously explored the use of echocardiography and cardiac magnetic resonance imaging (CMR) for NEN cardiac metastases.^25,26^ Echocardiography is routinely employed in NEN patients to assess for carcinoid valvular heart disease, although it has very limited role for the detection and characterization of cardiac metastases.^27^ CMR provides high spatial and contrast resolution for the assessment of cardiac tissue, as well as detailed information about valvular function and structure,^27^ and has been shown to demonstrate NEN cardiac metastases that were not identifiable on echocardiography.^28^

The optimal imaging strategy for the detection and characterization of NEN cardiac metastases remains unclear. An ENETS consensus guideline states that CMR should be performed in NEN patients in whom there is suspicion of cardiac metastases.^12^ It notes that CMR is superior to echocardiography in the detection and quantification of cardiac metastases, and that nuclear imaging often cannot provide definitive anatomical localization.^12^

We performed a single-center retrospective study to explore the role of SSTR PET/CT and CMR in NEN patients with cardiac metastases, to determine the degree of concordance between the findings of each modality, and to examine the advantages and disadvantages of each imaging technique. A secondary aim was to determine the safety of PRRT for patients with cardiac metastases in the subset of patients treated with this modality.

## Methods

A retrospective audit of patients presented at The Royal Brisbane and Women’s Hospital Neuroendocrine Tumour Multidisciplinary Team Meeting was performed. Consecutive patients were identified between January 2015 and May 2020 from the QOOL database (a clinical oncology database which integrates data from multiple sources). A low-risk ethics application was submitted to the Metro North Human Research Ethics Committee with ethics approval waived due to the retrospective nature of this study and the negligible risk to patients.

Inclusion criteria were patient age ≥ 18 years, histologically confirmed neuroendocrine neoplasm, at least one cardiac metastasis present on any imaging modality, information available regarding patient symptoms and treatment, and imaging results available for review on the hospital’s electronic medical record.

### SSTR PET/CT

Gallium 68-tetraazacyclododecane-tetraacetic acid-octreotate ([^68^Ga]Ga-DOTATATE) PET/CT scans were performed on a mCT Biograph scanner or mCT VISION scanner (Siemens Medical Solutions, Munich) from vertex to thighs. Low-dose non-contrast CT was also performed (100 keV, 80 mAs), reconstructed with a soft tissue kernel to a slice thickness of 2 mm for the purpose of PET attenuation correction and anatomical localization.

PET images were acquired 45-75 minutes post injection of [^68^Ga]Ga-DOTATATE, activity in the range of 120-200 MBq (3.2-5.4 mCi). PET scans were acquired in three-dimensional mode (matrix 400 × 400 or 440 × 440) using either multi-bed or FlowMotion (Siemens Medical Solutions, Munich). A bed equivalent time of 2-2.5 minutes was used with 4-5 minutes over the liver. The emission data was corrected for randoms, dead time, scatter, and attenuation, and was reconstructed iteratively using ordered-subsets expectation maximization (2 iterations 21 subsets on Biograph, or 5 iterations 5 subsets on VISION) with time of flight and point-spread function resolution recovery, followed by a post-reconstruction smoothing Gaussian filter.

In patients with multiple [^68^Ga]Ga-DOTATATE PET/CT scans performed during the audit period the scan performed closest in time to the CMR was analyzed. PET reporting was blinded; the reporters understood that only patients with cardiac metastases were included, however they did not know which imaging modality detected the metastases, did not have access to the clinical PET/CT report, previous imaging studies and reports (including CMR), or any other component of the patient clinical history.

Each [^68^Ga]Ga-DOTATATE PET/CT was re-reviewed by a nuclear medicine physician (12 years experience) and a nuclear medicine radiologist (4 years experience). Each reporter used Syngo Via (Siemens Medical Solutions, Munich) to view studies in standard anatomical format and to re-orient the cardiac images in standard cardiac formats (short axis, vertical long axis, horizontal long axis) based on the low-dose non-contrast CT. The left ventricular myocardial abnormalities were localized based on the standard American Heart Association (AHA) 17-segment system.^29^ The location of cardiac lesions located outside of the left ventricular myocardium were described with a text description. The SUV_max_ of each cardiac metastasis was measured, and the SUV_max_ of normal liver was also measured so that each cardiac metastasis could be classified as having a Krenning score of < 3 (SUV_max_ less than or equal to liver) or ≥ 3 (SUV_max_ greater than liver).^30^ In patients with multiple avid cardiac metastases, the four most avid lesions were analyzed. The results for lesion location and SUV_max_ for each reporter were tabulated separately and if discordant were reviewed by a third nuclear medicine physician (12 years experience) who resolved any discrepancies after reviewing the PET/CT images.

### Cardiac MRI

CMR scans were performed on a variety of different systems at several sites, a median of 44 days from SSTR PET/CT (range 3-220 days). The majority were acquired on a Siemens 3T platform. A typical protocol included steady state free precession cardiac cine images acquired prior to intravenous gadolinium for ventricular volumetric and valvular assessment, and to demonstrate the hemodynamic consequences from a cardiac mass. Imaging planes included 2-chamber, 3-chamber, and 4-chamber cines and a left ventricular short-axis cine stack. Tissue characterization of any mass with T1-weighted and T2-weighted imaging, along with fat saturation, was performed prior to intravenous gadolinium administration. First pass perfusion imaging and delayed enhancement sequences, including phase sensitive inversion recovery sequences, were acquired.

Each CMR was re-reviewed by an experienced imaging cardiologist (17 years experience), who was blinded to the SSTR PET/CT. Lesions were localized using the same standard AHA imaging segments as used for SSTR PET/CT.^29^

## Results

### Patients and tumor characteristic

19 patients with NENs with cardiac metastases identified on any form of imaging were identified with baseline characteristics outlined in Table 1. The median age of patients was 63 (range 43-72). Most patients had primary small bowel NENs (15/19, 79%), and the remainder had pulmonary NENs (4/19, 21%). The world health organisation (WHO) NEN tumor grade was available in 13/19 patients, with 8 grade 1 and 5 grade 2 primary tumors. There were no grade 3 tumors or neuroendocrine carcinomas. Of the patients 6/19 patients with unknown WHO NEN grade, all behaved clinically as low-grade NENs with a prognosis of 6 or more years after initial diagnosis with metastatic disease. Most patients also had liver metastases (15/19, 79%), and other sites of metastatic disease in order of frequency were nodal (12/19, 63%), skeletal muscle (8/19, 50%), bone (6/19, 31%), peritoneal (3/19, 16%), and pleural/pulmonary (3/19, 16%). No patients had carcinoid valvular heart disease, and only 2/19 patients had a cardiac lesion identified on echocardiogram. No patients had dedicated cardiac CT. 15 patients were treated with a somatostatin analogue, and 14 received PRRT (2 prior to the diagnosis of cardiac metastases, and 12 with known cardiac metastases). One patient required cardiac surgery (excision of a cardiac metastasis which was causing right ventricular outflow tract obstruction).

**Table 1:** Baseline patient characteristics

Patient/tumor characteristic	N (%) (n = 19)
Gender	
Male	11 (58%)
Female	8 (42%)
Age (years)	
30–50	1 (5%)
50–70	16 (84%)
≥ 70	2 (11%)
Location of primary NEN	
Small bowel	15 (79%)
Pulmonary	4 (21%)
WHO classification	
Grade 1 NET	8 (42%)
Grade 2 NET	5 (26%)
Grade 3 NET	0 (0%)
NEC	0 (0%)
Unknown	6 (32%)
Carcinoid valvular heart disease	
Present	0 (0%)
Absent	19 (100%)
Sites of metastatic disease (in addition to cardiac)	
Liver	15 (79%)
Peritoneal	3 (16%)
Pleura/pulmonary	3 (16%)
Lymph nodes	12 (63%)
Bone	6 (31%)
Muscular	8 (50%)
Management	
SSA alone	4 (21%)
PRRT + SSA	11 (58%)
PRRT alone	1 (5%)
Other	3 (16%)

### SSTR PET/CT

All 19 patients had [^68^Ga]Ga-DOTATATE PET/CT, and all 19 patients had avid cardiac metastases identified on [^68^Ga]Ga-DOTA-TATE PET/CT. 8/19 patients had one avid cardiac metastasis, 2/19 had two, 4/19 had three, 1/19 had four, and 4/19 had more than four.

44 [^68^Ga]Ga-DOTA-TATE avid cardiac metastases were analyzed in total. The median SUV_max_ was 7.5 (range 2.0-137.8). 22/44 cardiac metastases had a Krenning score ≥ 3, and 22/44 had a Krenning score < 3. The commonest location for metastases was within the left ventricle (26/44), followed by the right ventricle (10/44), interatrial septum (4/44), and other sites (4/44; pericardium, papillary muscle, atria). Exact lesion localization was difficult on PET/CT, with multiple AHA segments reported for most lesions (with reporters noting anatomical uncertainty rather than true segment overlap).

### Cardiac MRI

10/19 patients had CMR. At least one cardiac metastasis was identified in 9/10 patients who had CMR, and 14 metastases were identified for analysis on CMR (compared to 25 metastases in the same 10 patients on SSTR PET/CT). 5/10 patients had fewer metastases identified on CMR than SSTR PET/CT (including one patient with no cardiac metastasis identified on CMR), and in 5/10 patients all metastases were identified on both modalities. Lesion localization was more confident with CMR, with single AHA segments reported in all cases. In one case, CMR re-classified a metastasis thought to be in the interatrial septum on SSTR PET/CT as pericardial in location.

### Clinical impact of cardiac metastases

1/19 patients required cardiac surgery, with excision of a right ventricular outflow tract obstructing metastasis (a grade 1 NEN on histology) prior to PRRT.

12/19 patients with cardiac metastases proceeded to treatment with PRRT ([^177^Lu]Lu-DOTATATE). The presence of cardiac metastases did not preclude any patients from receiving PRRT, did not lead to reduced administered [^177^Lu]Lu-DOTATATE activity, and no major cardiac adverse events from PRRT were recorded. Specifically there was no evidence of cardiac failure or myocardial rupture, effusion or arrhythmia on routine post-therapy clinical assessment, serial post-therapy SPECT/CT or ECGs respectively. 9/12 patients received the prescribed full course of PRRT (four induction cycles for 8 patients, two re-induction cycles for 1 patient). Of the remaining 3/12 patients, 2 received only three of the four planned cycles due to thrombocytopenia and 1 patient moved interstate during treatment. The median cumulative activity was 31.9 GBq (862 mCi), range 16.5-48.0 GBq (446-1297 mCi), over a median of 4 cycles (range 2-6 cycles) (Table 2).

**Table 2:** Number and location of cardiac metastases identified on SSTR PET/CT and CMR

Patient	WHO grade (Ki-67, mitotic count per 10 high power fields)	SSTR PET/CT					Cardiac MRI			Cardiac surgery	PRRT
		Number of cardiac metastases identified	Lesion	Metastasis location	SUV_max_	Krenning Score ≥ 3	Number of days performed before/after PET	Number of cardiac metastases identified	Metastasis location		
1	G1 (1%, NA)	1	Lesion 1	LV segment 7/12	34.6	Y	+ 14	1	LV segment 7	N	N
2	NA (NA, NA)	2	Lesion 1	Interatrial septum	9.5	Y	NA	NA	NA	N	Y
			Lesion 2	Pericardium (inferior apical)	4.9	N			NA		
3	G2 (4.9%, 4)	1	Lesion 1	LV segment 8/9	9.7	Y	NA	NA	NA	N	N
4	G1 (< 1%, < 2)	1	Lesion 1	LV segment 2/3/8	40.1	Y	− 3	1	LV segment 2	N	Y
5	NA (NA, NA)	3	Lesion 1	LV segment 8/9/3	13.4	Y	− 20	1	LV segment 9	N	Y
			Lesion 2	Pericardial RV free wall	4.3	N			Not identified		
			Lesion 3	LV segment 13/RV free wall apex	2.9	N			Not identified		
6	G2 (3.2%, 6)	> 4	Lesion 1	LV segment 8/9/14	15.1	Y	+ 29	> 4	LV segment 14	N	Y
			Lesion 2	LV segment 2/3	12.8	Y			LV segment 3		
			Lesion 3	LV segment 1/2	10.5	Y			LV segment 11		
			Lesion 4	Lateral or anterior papillary muscle	8.8	Y			LV segment 12		
7	NA (NA, NA)	> 4	Lesion 1	RV free wall	5.6	N	+ 73	0	Not identified	N	Y
			Lesion 2	LV segment 5/10/11	5.3	N			Not identified		
			Lesion 3	RV insertion to LV segment 2/7/8	3.9	N			Not identified		
			Lesion 4	Pericardial recess/LA appendage	2	N			Not identified		
8	G2 (3.3%, NA)	1	Lesion 1	RV insertion to LV segment 8	3.7	N	NA	NA	NA	N	N*
9	G2 (5%, 2)	3	Lesion 1	RV insertion to LV segment 7/8	14	Y	+ 137	1	Right ventricle	N	Y
			Lesion 2	RV insertion/pericardial near LV segment 14	9.1	Y			Not identified		
			Lesion 3	LA/interatrial septum	8.2	Y			Not identified		
10	G1 (< 2%, NA)	1	Lesion 1	Interatrial septum	4.1	N	NA	NA	NA	N	Y
11	NA (NA, NA)	3	Lesion 1	LV segment 14/17	6.7	Y	NA	NA	NA	N	N*
			Lesion 2	Lateral to RA and within pericardium	4	N			NA		
			Lesion 3	LV segments 12/16	3	N			NA		
12	G1 (1–2%, NA)	4	Lesion 1	LV segment 13/14/17/RV insertion anterior wall	8.3	Y	NA	NA	NA	N	N
			Lesion 2	LV segment 8	5.6	N			NA		
			Lesion 3	LV segment 1	4.8	N			NA		
			Lesion 4	Basal RV wall/RVOT	2.7	N			NA		
13	G1 (< 1%, NA)	> 4	Lesion 1	LV segment 16/pericardium	17	Y	NA	NA	NA	N	Y
			Lesion 2	LV segment 1/6	4.6	N			NA		
			Lesion 3	LV segment 1/2	4.2	N			NA		
			Lesion 4	LV segment 7	2.9	N			NA		
14	G1 (NA, < 2)	3	Lesion 1	LV segment 7	22.6	Y	− 220	1	LV segment 7	N	Y
			Lesion 2	LV segment 8/9	3.2	N			Not identified		
			Lesion 3	RV papillary muscle/cavity (mid-basal)	2.3	N			Not identified		
15	G1 (< 1%, < 2)	1	Lesion 1	LV segment 3/4	9.1	N	+ 56	1	LV segment 3	N	N
16	G1 (< 2%, NA)	> 4	Lesion 1	RVOT/RV	137.8	Y	− 32	3	RVOT	Y	Y
			Lesion 2	LV segment 2	27.2	Y			LV segment 2		
			Lesion 3	LV segment 11/16	18.6	Y			LV segment 11		
			Lesion 4	LV segment 11/12/16	11.8	Y			Not identified		
17	NA (NA, NA)	1	Lesion 1	Interatrial septum	39.8	Y	+ 80	1	Pericardial	N	Y
18	G2 (5%, 2)	1	Lesion 1	Right ventricle insertion in anteroseptal wall at apical part	12.8	Y	NA	NA	NA	N	Y
19	NA (NA, NA)	2	Lesion 1	LV segment 13/7	2.6	N	NA	NA	NA	N	N
			Lesion 2	LV segment 13/14	2.3	N			NA		

*Previously had prior to appearance of cardiac metastases (PRRT).

## Discussion

**Figure 1:**
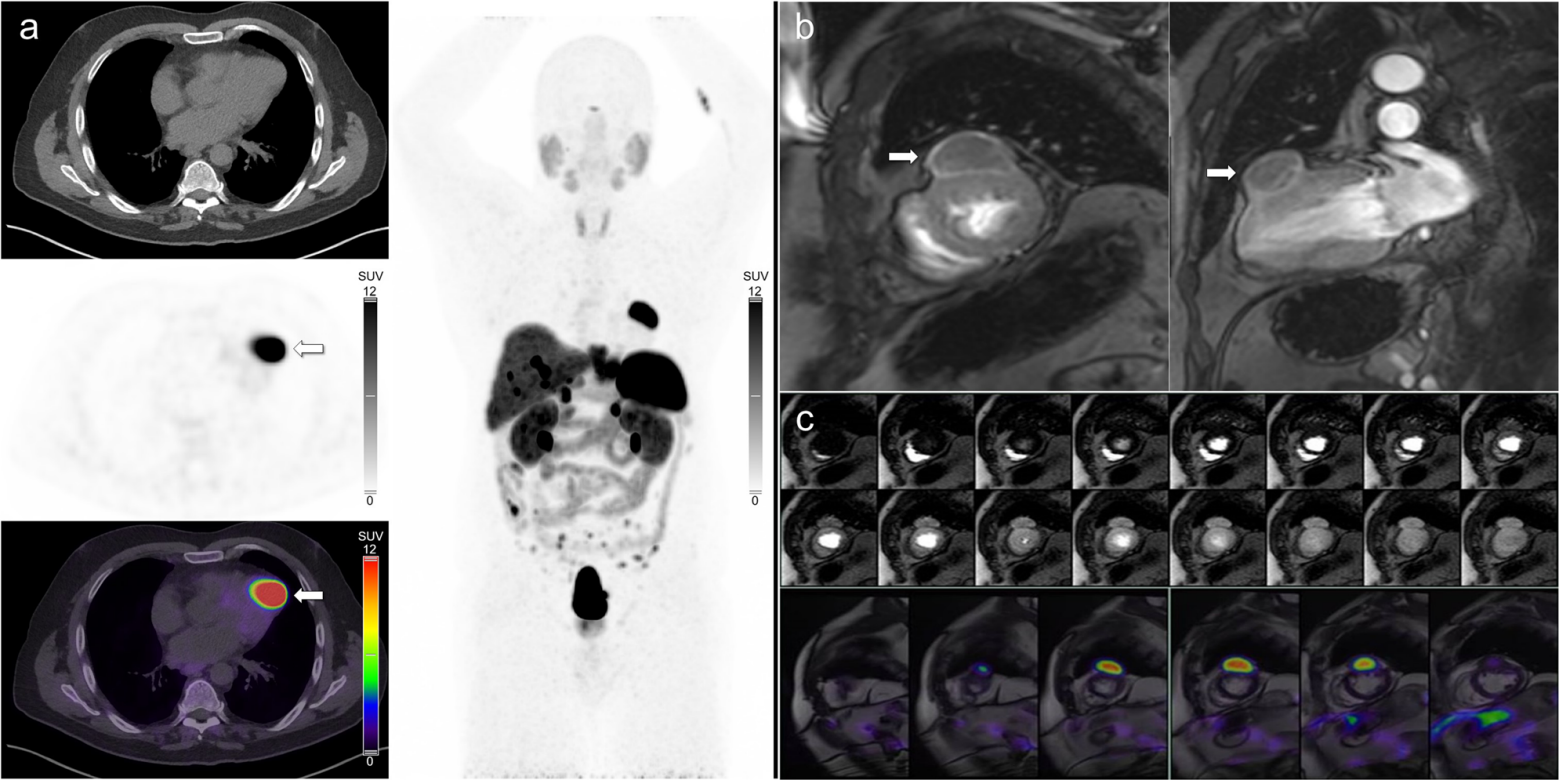
(**A**) [^68^Ga]Ga-DOTATATE PET/CT images in a patient with metastatic small bowel NEN demonstrating a large intensely avid metastasis in the mid anterior left ventricle (segment 7). Note the high target to background uptake and excellent lesion conspicuity. (**B**) Corresponding CMR images (T1-weighted delayed post-contrast short-axis and two-chamber views) demonstrate the same lesion. (**C**) Retrospectively fused short axis PET/MRI images of the same lesion

In this retrospective single-center study SSTR PET/CT identified all NEN cardiac metastases, including many that were not identified on CMR. SSTR PET/CT is highly sensitive for imaging most NEN lesions, and is recommended for tumor staging, preoperative imaging, and restaging.^10^ On SSTR PET/CT, lesions typically demonstrate very high target-to-background radiotracer uptake, facilitating excellent lesion conspicuity (Figure 1). A limitation of SSTR PET/CT is the difficulty in determining the exact location, size, and functional impact of cardiac metastases. This is due to multiple factors including the inherent spatial resolution limitations of PET, the relatively poor anatomical detail afforded by low-dose non-contrast CT, and the possibility of misregistration between PET and CT data due to respiratory and cardiac motion.

**Figure 2:**
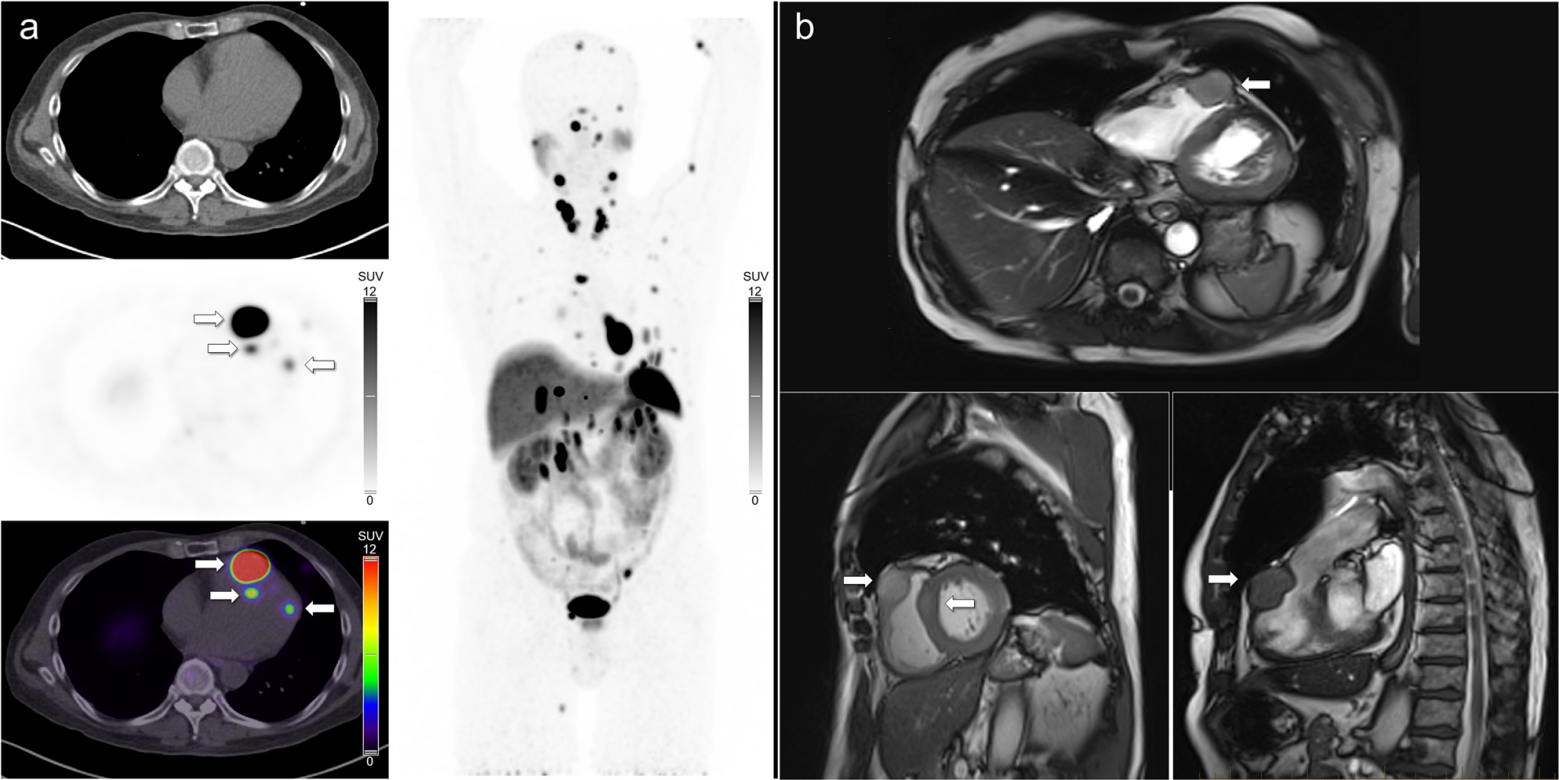
(**A**) [^68^Ga]Ga-DOTATATE PET/CT images in a patient with metastatic pulmonary NEN demonstrating a large intensely avid metastasis in the right ventricular outflow tract, with two other smaller avid metastases in the left ventricle. (**B**) CMR images (multiplanar steady-state free precession sequences) better characterize the size and functional significance of the right ventricular outflow metastasis just proximal to the pulmonary valve with luminal narrowing. One of the left ventricular lesions is subtly visible on the two-chamber view. The patient proceeded to surgical resection of the right ventricular outflow tract lesion prior to PRRT

**Figure 3:**
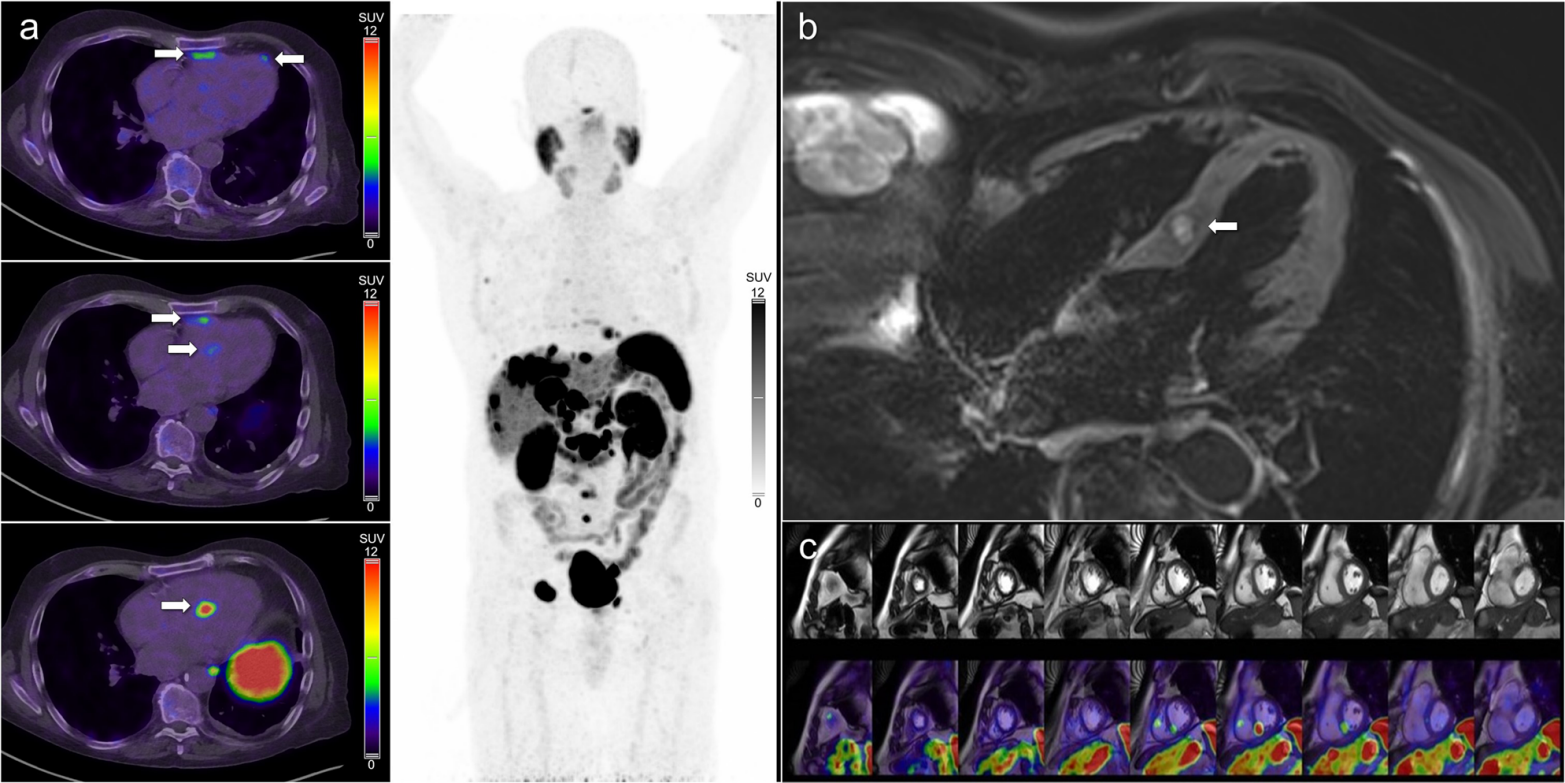
(**A**) [^68^Ga]Ga-DOTATATE PET/CT images in a patient with metastatic small bowel NEN demonstrating multiple avid cardiac metastases involving the left and right ventricle. (**B**) CMR T2-weighted spectral attenuated inversion recovery four-chamber image and (**C**) retrospectively fused PET/MRI short axis images demonstrate a single concordant metastasis in the mid inferoseptal left ventricle (segment 9). The other DOTATATE avid cardiac metastases were not evident on CMR

**Figure 4:**
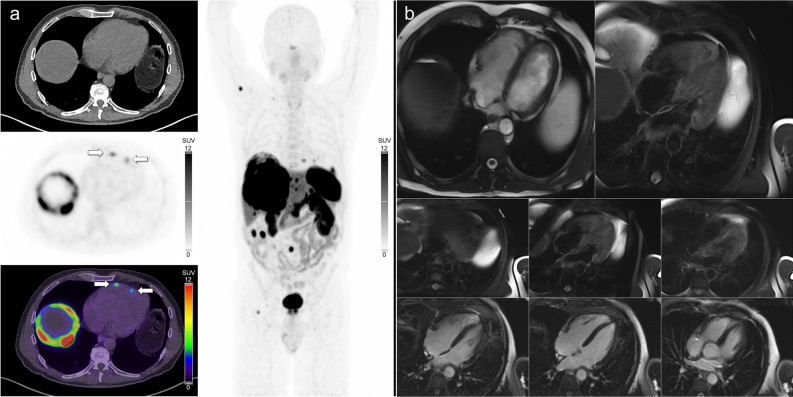
(**A**) [^68^Ga]Ga-DOTATATE PET/CT images in a patient with metastatic small bowel NEN demonstrating multiple avid cardiac metastases involving the right ventricular free wall. (**B**) CMR four-chamber images (T2-weighted, T2-weighted fat saturated, and late gadolinium enhancement sequences) do not demonstrate these metastases. No lesions were identifiable on CMR in this case

CMR is generally regarded as the gold-standard imaging technique for the characterization of cardiac lesions (including cardiac metastases) due to its high contrast resolution and excellent tissue characterization.^31^ In our study, CMR identified fewer metastases than SSTR PET/CT (Figures 2, 3), and did not identify any metastases that were not also detected on SSTR PET/CT. In one case CMR failed to identify any metastasis when many (> 4) were evident on SSTR PET/CT (Figure 4). Despite this, when a metastasis was detected, CMR was able to offer more accurate lesion localization. By providing a more accurate anatomical and morphological depiction of cardiac metastases, as well as interrogating the relationship to adjacent structures, CMR can determine the potential functional significance of a lesion and aid in surgical decision making and planning. In the one patient in our series who required surgery, CMR was vital in demonstrating the functional significance of the right ventricular outflow tract lesion and provided valuable surgical guidance (Figure 2). A further advantage of CMR is the lack of ionizing radiation compared to the radiation associated with PET/CT. Practical disadvantages of CMR include the need for intravenous gadolinium (which can be contraindicated in severe renal impairment), and the fact that MRI can be difficult or contraindicated in patients with some implantable devices; both factors which are not applicable to PET/CT. Patients also generally find PET/CT more tolerable than MRI, particularly if they are claustrophobic.^32,33^

Our findings suggest that there is a role for both SSTR PET/CT and CMR in the evaluation of NEN cardiac metastases, with SSTR PET/CT excelling in lesion detection, and CMR optimized for lesion characterization. In clinical practice we believe that SSTR PET/CT should generally be performed first, followed by referral for CMR if cardiac metastases are identified. This allows appropriately directed CMR sequences to be performed to further characterize the location, quantify the size, and define the extent of ventricular wall involvement of metastases, also providing a baseline for accurate comparisons over time. Direct comparison of SSTR PET/CT and CMR images appears to improve lesion identification on CMR and is strongly recommended. Software packages can be utilized to retrospectively fuse PET functional information with CMR detailed anatomy (Figures 2, 3), and the advantages of these hybrid approaches in NEN cardiac metastases have been previously reviewed.^34^ Combined PET/MRI scanners may provide improved image co-registration but both scans would rarely be indicated at the same time unless cardiac metastases were known prior to referral or specific detailed imaging co-registration was required. Although PET/MRI has the advantage of combining both tests into a single study, disadvantages include the inevitable compromise between examination time, number of sequences performed, and image quality (with reduced spatial resolution accompanying the need for large field of view whole body imaging), suboptimal evaluation of the pulmonary parenchyma, and limited clinical availability in many parts of the world.

Consistent with published literature,^16,18^ echocardiogram was highly insensitive in the detection of NEN cardiac metastases, only identifying cardiac metastases in 2/19 patients. This is likely due to multiple factors including poor tissue contrast, small lesion size, and operator dependence. Although it has a clearly established role in the detection of carcinoid valvular disease, we suggest that echocardiogram has a very limited role in the detection and assessment of NEN cardiac metastases, and if suspected SSTR PET/CT should be performed, with further characterization by CMR as required.

This is one of the larger case series on NEN cardiac metastases performed to date, and our findings are consistent with the results of prior studies. In keeping with the literature, there was no relationship between the presence of NEN cardiac metastases and carcinoid valvular heart disease, with no patients having both pathologies.^12^ Similarly, we confirmed prior reports that NEN cardiac metastases usually arise from well-differentiated small bowel NENs in advanced disease, with the most common metastasis location being the left ventricle.^16,35^ We noted a relatively high association with skeletal muscle metastases (present in half of our patient cohort with cardiac metastases); an association that has not been previously emphasized in the literature. Liu previously reported orbital metastases in 4/21 NEN patients with cardiac metastases,^16^ but did not specify if they were extra-ocular muscle metastases or metastases to other sites within the orbit. Our findings suggest a degree of tropism between cardiac and skeletal muscle in metastatic NEN.

The very high sensitivity of SSTR PET/CT for the detection of NEN cardiac metastases is concordant with the findings of Liu et al, who published a similar retrospective case series of 25 patients with NEN cardiac metastases identified on various imaging modalities.^16^ In their study, SSTR PET/CT was performed in 21 patients, and was similarly 100% sensitive for the detection of cardiac metastases. They noted that SSTR PET/CT may be more sensitive than CMR, which was performed in only 2 patients to further evaluate cardiac uptake on SSTR PET/CT, with concordant lesions identified in both cases. Advantages of our study were the partially blinded nature (whereby each imaging modality was interpreted without the results of the other), and the higher number of patients who had both SSTR PET/CT and CMR.

**Figure 5:**
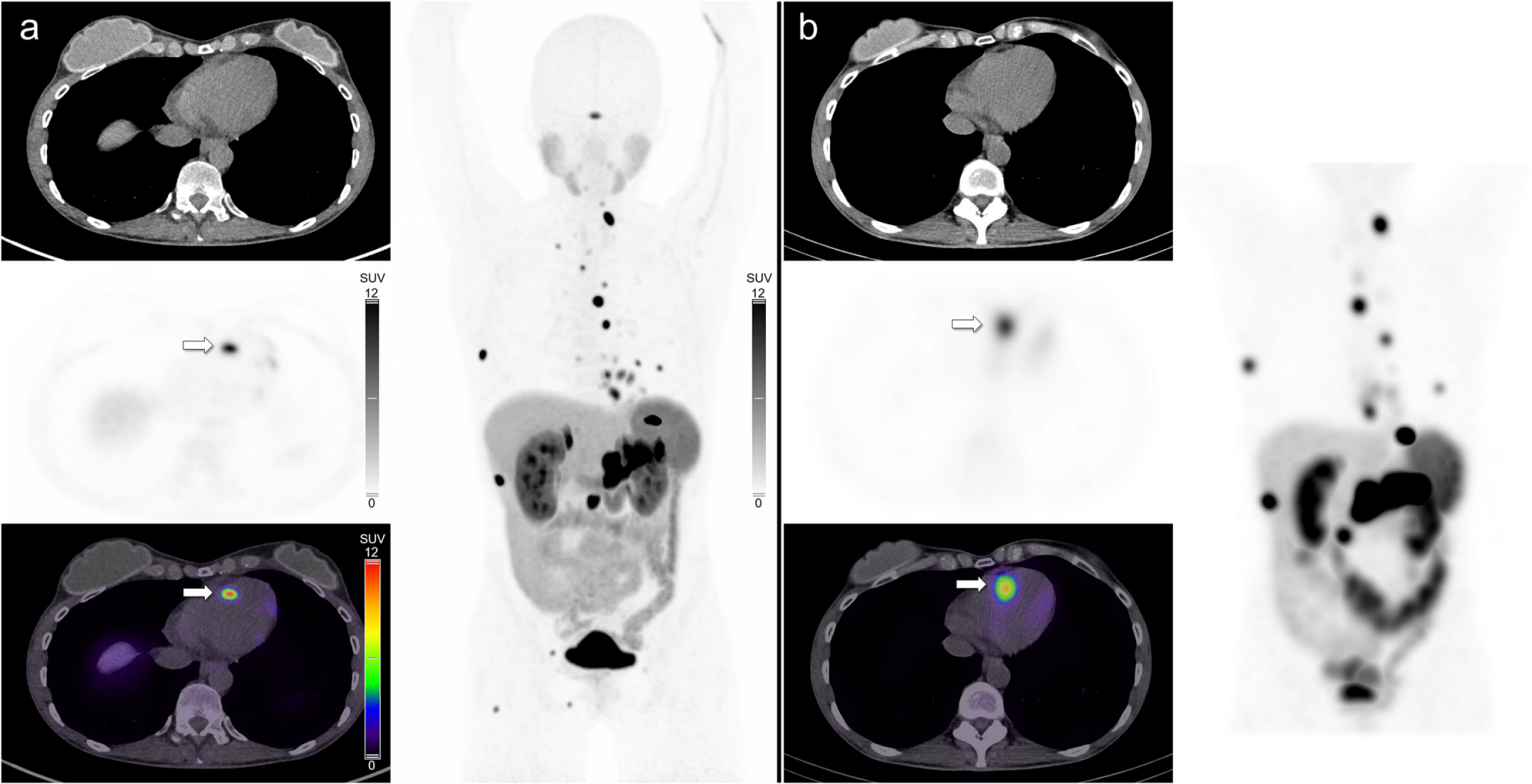
(**A**) [^68^Ga]Ga-DOTATATE PET/CT images in a patient with metastatic small bowel NEN demonstrating an intensely avid metastasis in the apical septal left ventricle (segment 14). (**B**) SPECT/CT images performed 24 hours following treatment with 8.3 Gbq (224 mCi) of intravenous [^177^Lu]Lu-DOTATATE demonstrate high radiopharmaceutical retention within this metastasis. No cardiac complication occurred

In our series PRRT was safely administered to 12 patients with NEN cardiac metastases (Figure 5), with no cardiac adverse events recorded. This is the second largest reported series of NEN patients with cardiac metastases treated with PRRT, and our findings supplement the small body of existing literature regarding the use of PRRT in this patient cohort.^16,20–24^ We suggest that PRRT can be safely performed in these patients without the need for dose reduction, noting that the only cardiac complication arising from PRRT in the setting of NEN cardiac metastases reported in the literature is pericarditis.^16^

The key limitations of this study are the retrospective nature and the relatively small sample size, in particular the small number of patients who had both SSTR PET/CT and CMR (although to our knowledge this is the largest study of patients having both imaging modalities to date). It is also possible that selection bias contributed to the apparent superior sensitivity of SSTR PET/CT, as SSTR PET/CT is performed in almost all NEN patients, while CMR is a non-routine investigation in the oncology setting, usually reserved for characterizing a finding identified on another imaging modality (generally SSTR PET/CT). Similarly, it is possible that the temporal gap between the two imaging modalities (SSTR PET/CT and CMR) could have impacted the results. Larger prospective trials are therefore required to validate the findings. Furthermore, multiple CMR studies were performed externally, while all SSTR PET/CT scans were performed at our tertiary institution. It should be noted that unlike PET/CT, CMR requires a tailored imaging protocol for optimal detection and characterization of cardiac masses. Such protocols are often heterogeneous across sites and scanners (far more heterogeneous than PET/CT protocols), and in some cases suboptimal. It is therefore possible that this also contributed to the lower sensitivity of CMR in this study.

## New Knowledge Gained

- SSTR PET/CT appears more sensitive than CMR for detection of NEN cardiac metastases, but CMR provides more accurate localization.
- PRRT can be safely administered in patients with NEN cardiac metastases.
- There may be an association between NEN cardiac metastases and skeletal muscle metastases.

## Conclusion

The findings of this retrospective single-center study suggest that SSTR PET/CT and CMR are complementary investigations in the imaging of NEN cardiac metastases, while echocardiogram has a very limited role. Although SSTR PET/CT appears to have higher sensitivity for detection, CMR provides more accurate lesion characterization regarding the exact location, size, and functional significance of metastases. Both investigations offer useful information for the planning of treatment including PRRT, which was administered safely in a multidisciplinary setting without the need for dose reduction.

### Supplementary Information

Below is the link to the electronic supplementary material.

Supplementary file1 (PPTX 778 KB)

## Data Availability

Not available.

## Abbreviations

AHA: American Heart Association
CMR: Cardiac magnetic resonance imaging
ENETS: European Neuroendocrine Tumour Society
GBq: Gigabecquerel
MBq: Megabecquerel
mCi: Millicurie
NEN: Neuroendocrine neoplasm
PRRT: Peptide receptor radionuclide therapy
SSTR PET/CT: Somatostatin receptor positron emission tomography/computed tomography
SUV_max_: Maximum standardised uptake value
