# Supplementary material for: Cardiac metastases from neuroendocrine neoplasms: complementary role of SSTR PET/CT and cardiac MRI
Source: J Nucl Cardiol. 2023 Aug 16;30(6):2676–91. doi: 10.1007/s12350-023-03345-w (PMC10682059; doi:10.1007/s12350-023-03345-w)
Supplement: Supplementary file 1 — Supplementary file1 (PPTX 778 KB) [file 12350_2023_3345_MOESM1_ESM.pptx]

## Slide 1
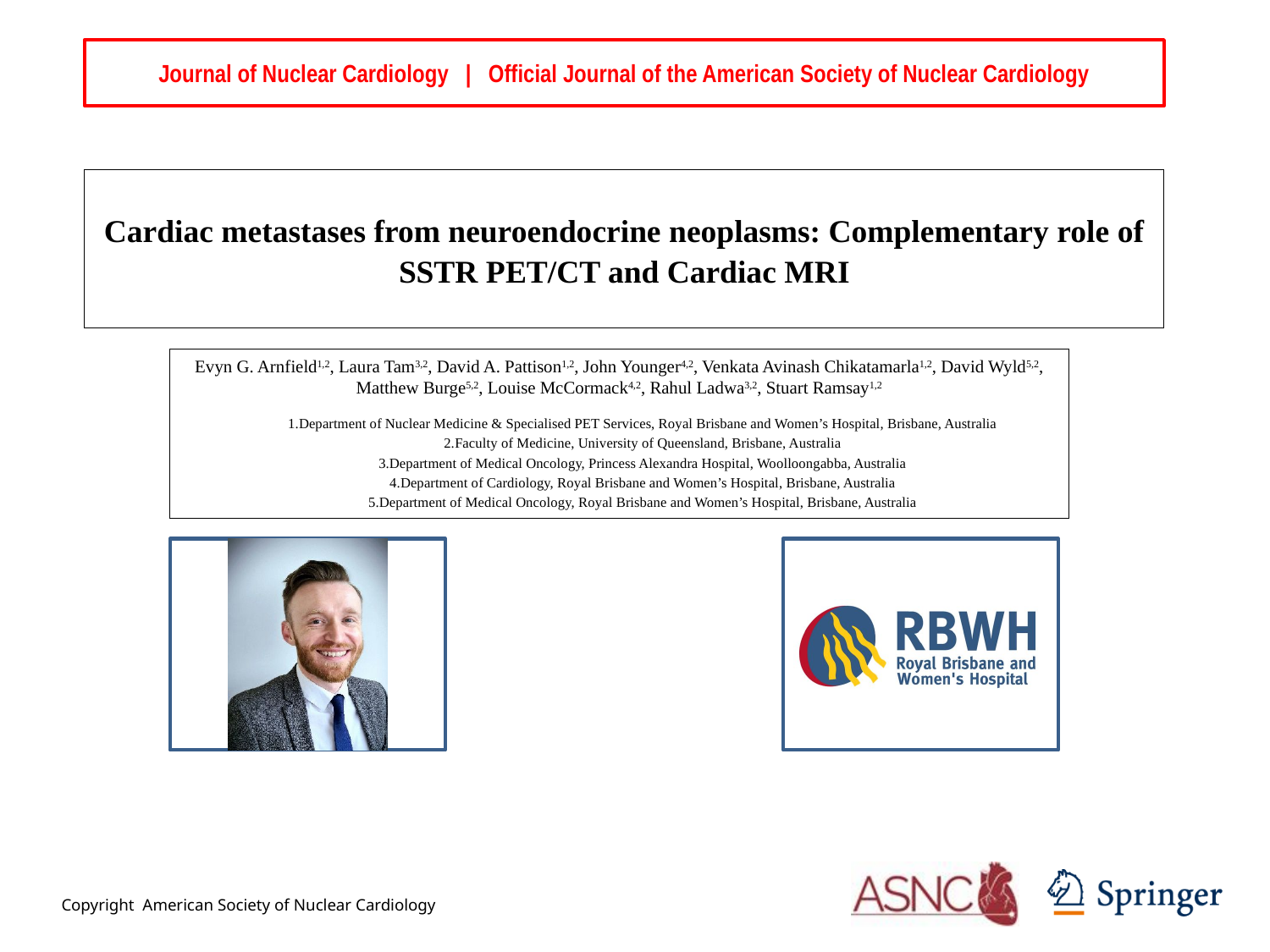

Journal of Nuclear Cardiology | Official Journal of the American Society of Nuclear Cardiology
# Cardiac metastases from neuroendocrine neoplasms: Complementary role of SSTR PET/CT and Cardiac MRI
Evyn G. Arnfield1,2, Laura Tam3,2, David A. Pattison1,2, John Younger4,2, Venkata Avinash Chikatamarla1,2, David Wyld5,2, Matthew Burge5,2, Louise McCormack4,2, Rahul Ladwa3,2, Stuart Ramsay1,2
Department of Nuclear Medicine & Specialised PET Services, Royal Brisbane and Women’s Hospital, Brisbane, Australia
Faculty of Medicine, University of Queensland, Brisbane, Australia
Department of Medical Oncology, Princess Alexandra Hospital, Woolloongabba, Australia
Department of Cardiology, Royal Brisbane and Women’s Hospital, Brisbane, Australia
Department of Medical Oncology, Royal Brisbane and Women’s Hospital, Brisbane, Australia
Copyright American Society of Nuclear Cardiology

## Slide 2
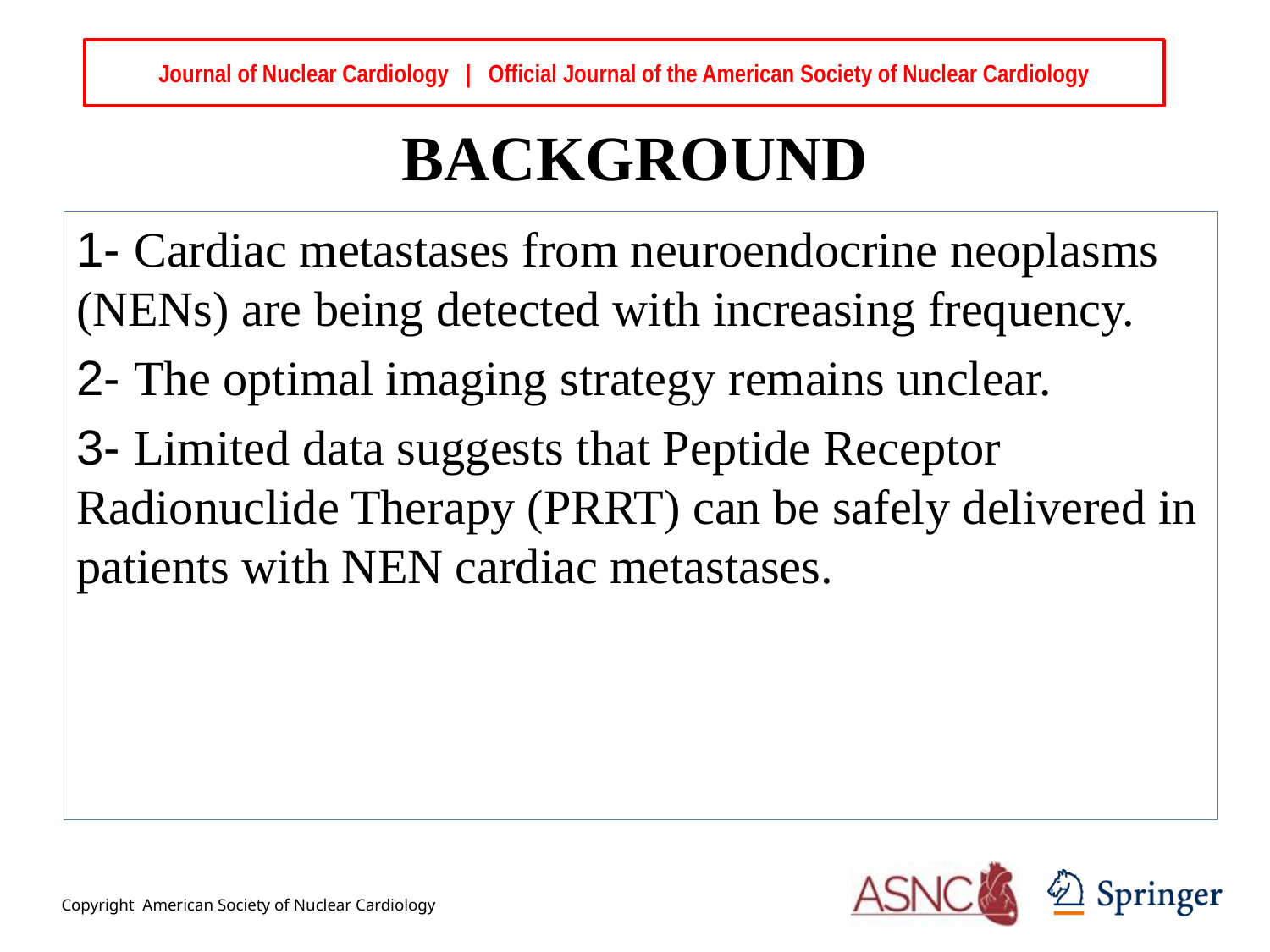

Journal of Nuclear Cardiology | Official Journal of the American Society of Nuclear Cardiology
# BACKGROUND
1- Cardiac metastases from neuroendocrine neoplasms (NENs) are being detected with increasing frequency.
2- The optimal imaging strategy remains unclear.
3- Limited data suggests that Peptide Receptor Radionuclide Therapy (PRRT) can be safely delivered in patients with NEN cardiac metastases.
Copyright American Society of Nuclear Cardiology

## Slide 3
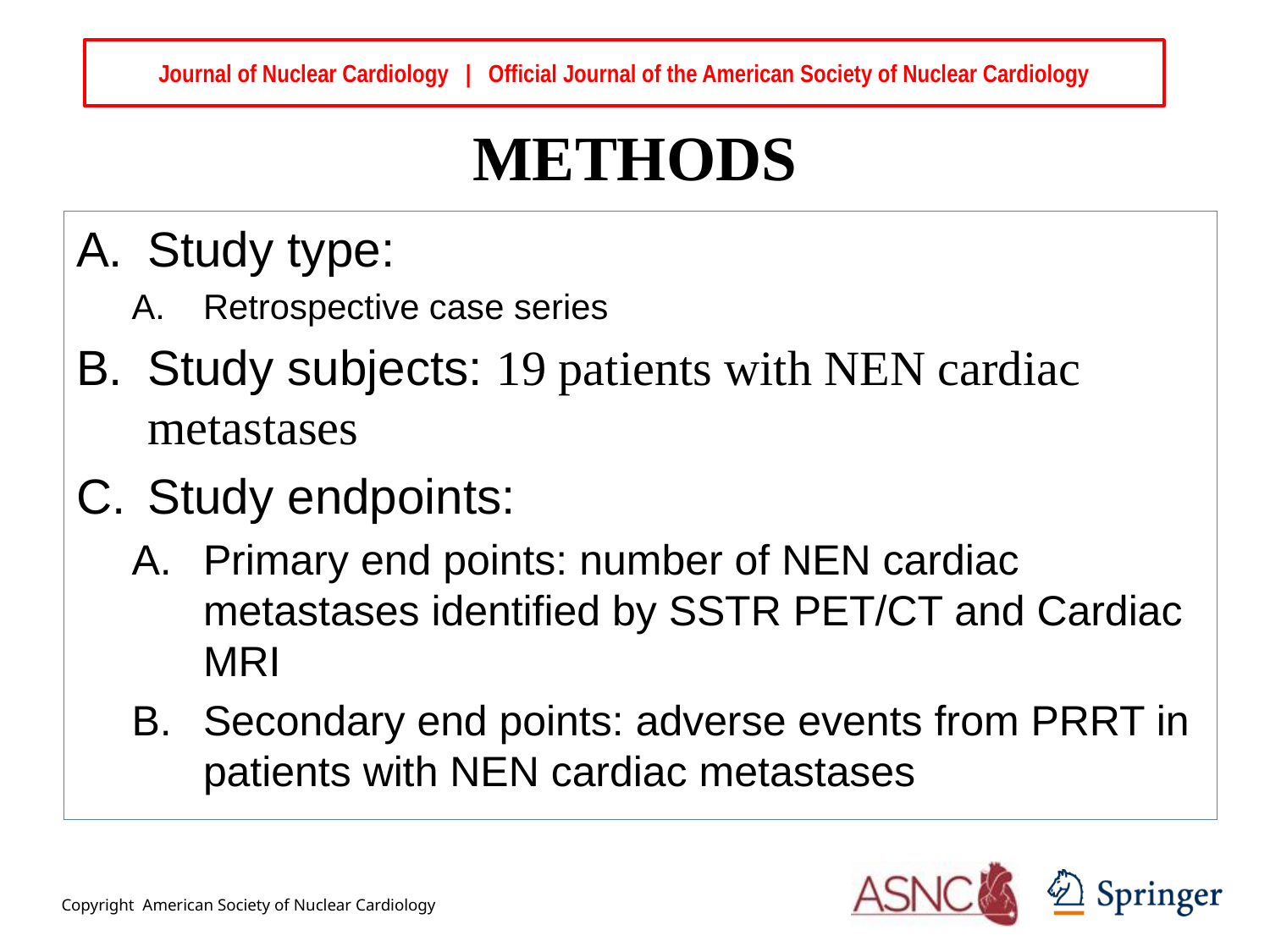

Journal of Nuclear Cardiology | Official Journal of the American Society of Nuclear Cardiology
# METHODS
Study type:
Retrospective case series
Study subjects: 19 patients with NEN cardiac metastases
Study endpoints:
Primary end points: number of NEN cardiac metastases identified by SSTR PET/CT and Cardiac MRI
Secondary end points: adverse events from PRRT in patients with NEN cardiac metastases
Copyright American Society of Nuclear Cardiology

## Slide 4
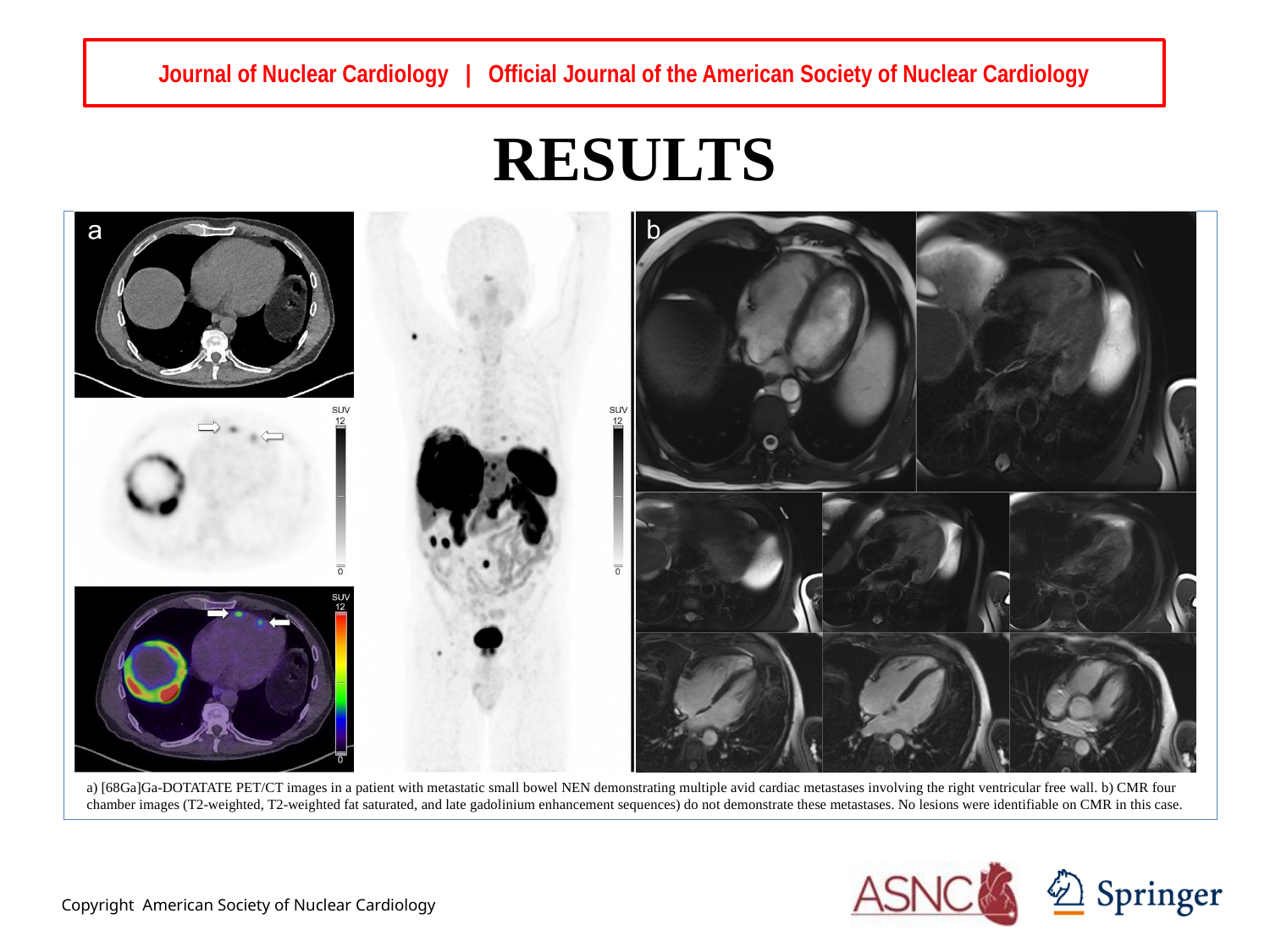

Journal of Nuclear Cardiology | Official Journal of the American Society of Nuclear Cardiology
# RESULTS
a) [68Ga]Ga-DOTATATE PET/CT images in a patient with metastatic small bowel NEN demonstrating multiple avid cardiac metastases involving the right ventricular free wall. b) CMR four chamber images (T2-weighted, T2-weighted fat saturated, and late gadolinium enhancement sequences) do not demonstrate these metastases. No lesions were identifiable on CMR in this case.
Copyright American Society of Nuclear Cardiology

## Slide 5
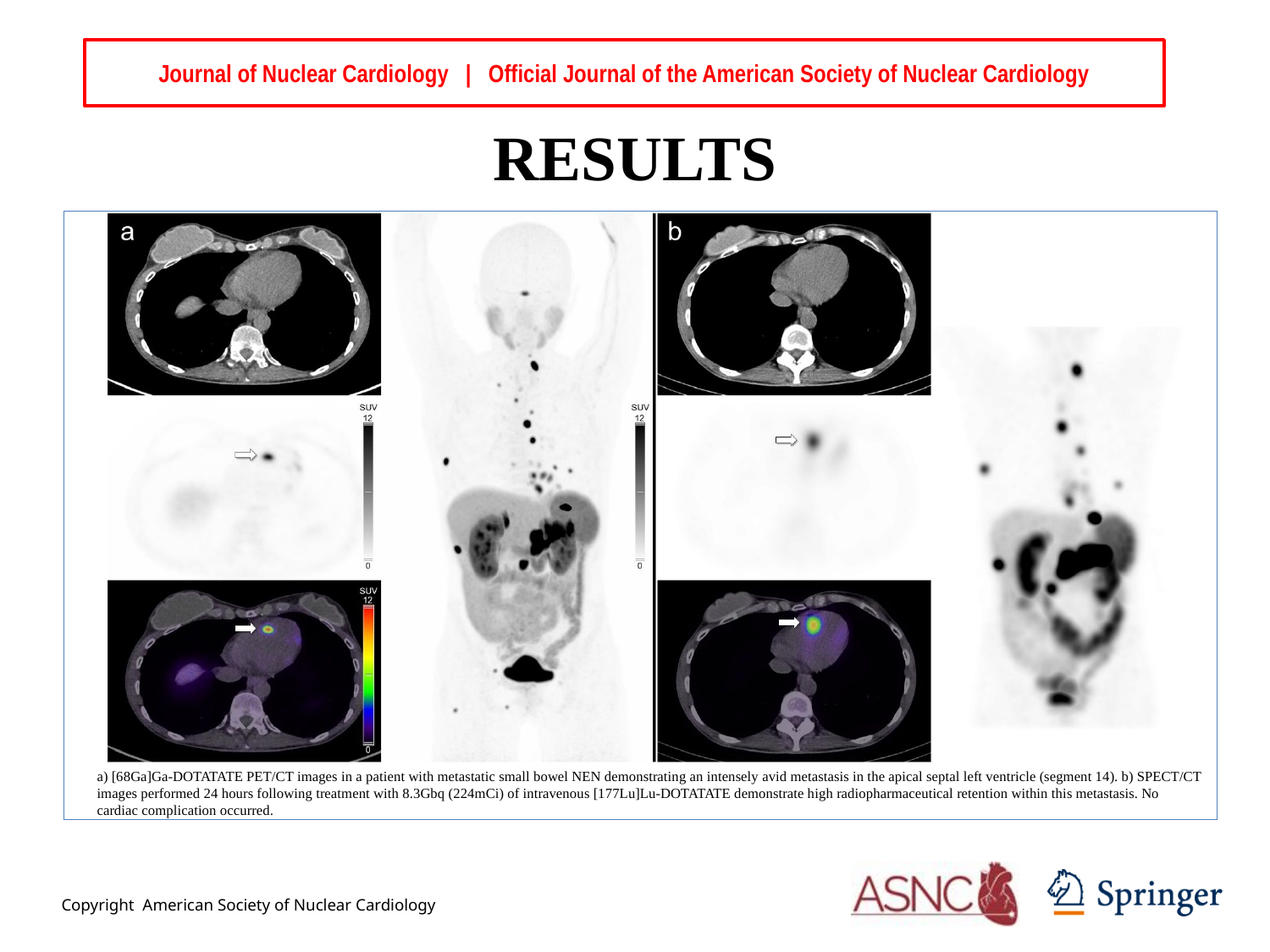

Journal of Nuclear Cardiology | Official Journal of the American Society of Nuclear Cardiology
# RESULTS
a) [68Ga]Ga-DOTATATE PET/CT images in a patient with metastatic small bowel NEN demonstrating an intensely avid metastasis in the apical septal left ventricle (segment 14). b) SPECT/CT images performed 24 hours following treatment with 8.3Gbq (224mCi) of intravenous [177Lu]Lu-DOTATATE demonstrate high radiopharmaceutical retention within this metastasis. No cardiac complication occurred.
Copyright American Society of Nuclear Cardiology

## Slide 6
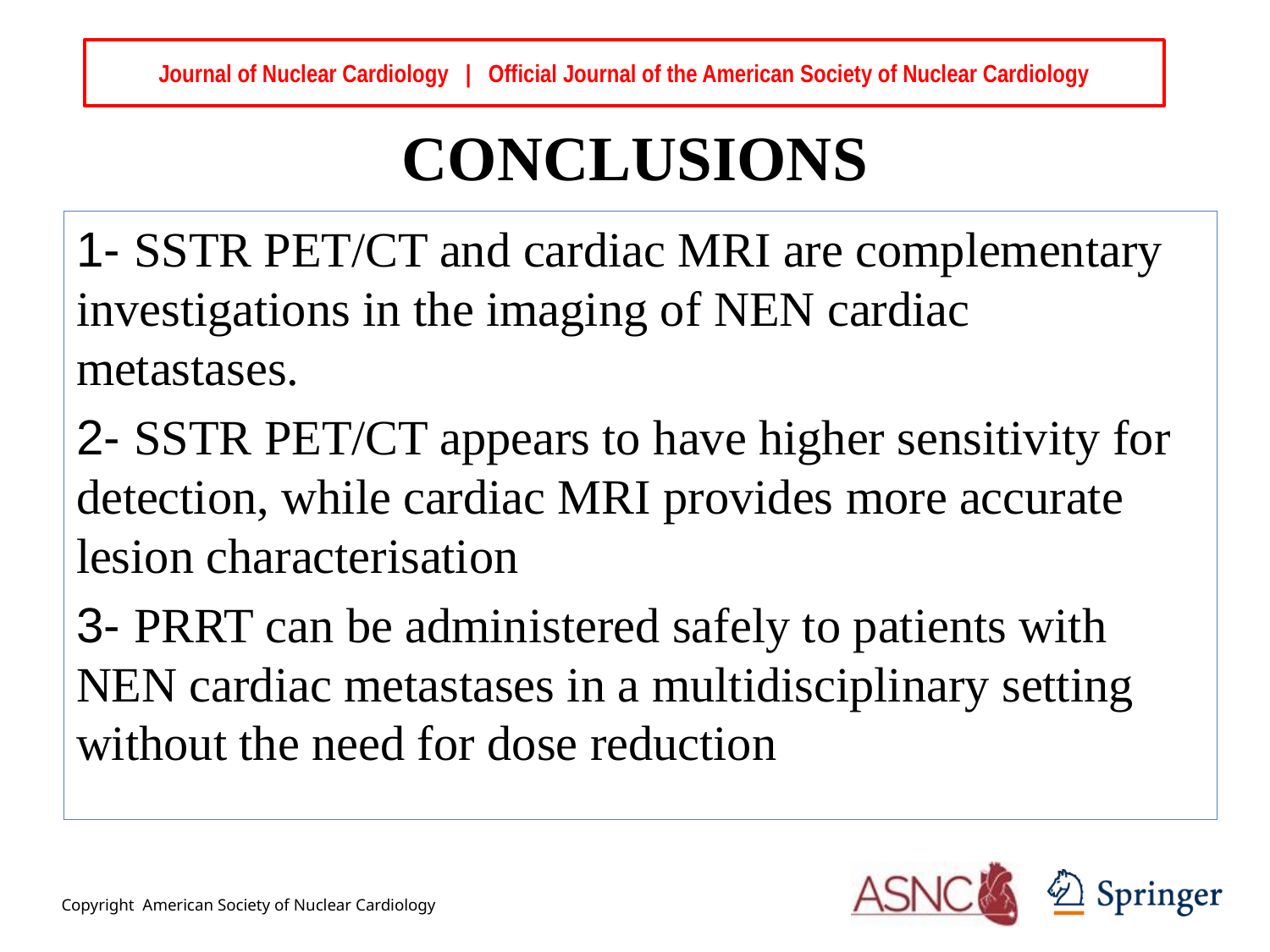

Journal of Nuclear Cardiology | Official Journal of the American Society of Nuclear Cardiology
# CONCLUSIONS
1- SSTR PET/CT and cardiac MRI are complementary investigations in the imaging of NEN cardiac metastases.
2- SSTR PET/CT appears to have higher sensitivity for detection, while cardiac MRI provides more accurate lesion characterisation
3- PRRT can be administered safely to patients with NEN cardiac metastases in a multidisciplinary setting without the need for dose reduction
Copyright American Society of Nuclear Cardiology
